# A GA-Based Approach to Hide Sensitive High Utility Itemsets

**DOI:** 10.1155/2014/804629

**Published:** 2014-03-03

**Authors:** Chun-Wei Lin, Tzung-Pei Hong, Jia-Wei Wong, Guo-Cheng Lan, Wen-Yang Lin

**Affiliations:** ^1^Innovative Information Industry Research Center, Harbin Institute of Technology Shenzhen Graduate School, Shenzhen 518055, China; ^2^Shenzhen Key Laboratory of Internet Information Collaboration, Harbin Institute of Technology Shenzhen Graduate School, Shenzhen 518055, China; ^3^Department of Computer Science and Information Engineering, National University of Kaohsiung, Kaohsiung 811, Taiwan; ^4^Department of Computer Science and Engineering, National Sun Yat-sen University, Kaohsiung 804, Taiwan; ^5^Department of Mathematics and Computer Sciences, Fuqing Branch of Fujian Normal University, Fuzhou, Fujian 350300, China

## Abstract

A GA-based privacy preserving utility mining method is proposed to find appropriate transactions to be inserted into the database for hiding sensitive high utility itemsets. It maintains the low information loss while providing information to the data demanders and protects the high-risk information in the database. A flexible evaluation function with three factors is designed in the proposed approach to evaluate whether the processed transactions are required to be inserted. Three different weights are, respectively, assigned to the three factors according to users. Moreover, the downward closure property and the prelarge concept are adopted in the proposed approach to reduce the cost of rescanning database, thus speeding up the evaluation process of chromosomes.

## 1. Introduction

Data mining techniques are used to mind the useful information and knowledge from various databases [1–9] to aid managers for making the efficient decisions. Due to the quick proliferation of electronic data in the government, the corporation, and the organization, the mined knowledge may, however, implicitly contain confidential information and lead to privacy threats if they are misused. Many algorithms were thus proposed for hiding the sensitive itemsets in privacy preserving data mining (PPDM) [10–12]. The privacy preserving utility mining (PPUM) is an extension of PPDM, considering the utility factors in the transactions, such as the quantities and profits. Yeh and Hsu firstly proposed the protection algorithm to modify the quantities of items for hiding sensitive high utility itemsets [13]. In the privacy preserving issue for data sanitization, however, it is not suitable to modify the quantities of the items in the transactional database.

Recently, genetic algorithms (GAs) [14, 15] have become an important research issue since they could provide feasible solutions in a limited amount of time. It is derived from the evolutionary ideas of natural selection and genetics, including the selection, crossover, and mutation operations to evaluate the chromosomes in the populations for producing the appropriate solutions. In this paper, a GA-based approach through transaction insertion is thus proposed to hide the sensitive high utility itemsets in privacy preserving utility mining. A flexible evaluation function with three factors is designed and three artificial weights are, respectively, assigned to three factors according to users. The maximal inserted utility for transaction insertion is found to define the number of genes in a chromosome. The proposed approach adopted the downward closure property in utility mining [16] and the prelarge concept [17] to reduce computations for rescanning the original database in evaluation process. To the best of our knowledge, this is the first paper to address the privacy preserving high utility itemsets mining based on genetic algorithms. The contributions of this paper can be illustrated as below.

1. We adopt a GA-based algorithm to sanitize the original database in order to hide sensitive high utility itemsets through transaction insertion. To the best of our knowledge, there is no GA-based approach to perturb the original database for data sanitization through transaction insertion in PPUM.
2. We adopt prelarge concepts to reduce the execution time for rescanning the original database in chromosome evaluation.
3. We design a novel evaluation function to consider the side effects in PPUM such as hiding failures, missing costs, and artificial costs. The adjustable weights are also considered for the three side effects according to users.

## 2. Review of Related Works

In this section, the related works are then described below. They are high utility mining, prelarge concepts, data sanitization, and genetic algorithms.

### 2.1. High Utility Mining

Utility mining [16, 18], an extension of frequent itemset mining, is based on the measurement of local transaction utility and external utility. The utility of an item in a transaction is defined as the product of its quantity multiplied by its profit. The utility of an itemset in a transaction is the sum of the utilities of all items in this transaction. If the ratio of the utilities for an itemset in all transactions is larger than or equal to the user-specified minimum high utility threshold, the itemset is thus considered as a high utility itemset.

In the past, many algorithms were proposed for mining high utility itemsets. Yao and Hamilton proposed an algorithm for efficiently mining high utility itemsets based on mathematical property of utility constraints [18]. Two pruning strategies were used to reduce the search space based on the utility upper bounds and expected utility upper bounds, respectively. Liu et al. proposed a two-phase algorithm for efficiently discovering high utility itemsets [16] based on downward closure property. The proposed algorithm consists of two phases to level wisely generate-and-test high utility itemsets to, respectively, find the effective upper bound of each candidate itemset in the transactions according to the downward closure property of transaction-weighted utilization and then perform an additional database scan to find the real utility values of the remaining candidate itemsets for discovering high utility itemsets. Lin et al. presented a high utility pattern (HUP)-tree [19] for efficiently mining the high utility itemsets.

### 2.2. Prelarge Concept

**Figure 1:**
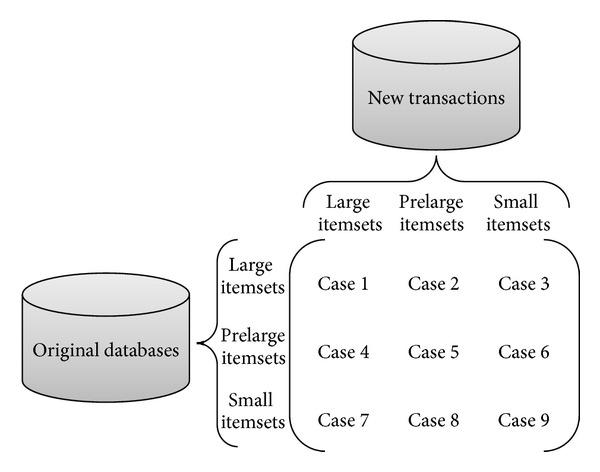
Nine cases when new transactions are inserted into existing databases.

In real-world applications, it is necessary to maintain the discovered knowledge in dynamic database, thus reducing the rescanning time of original database. Hong et al. proposed prelarge concept for efficiently maintaining the discovered information in dynamic databases [17]. A prelarge itemset is not really large (frequent) but might become large (frequent) in the future through data insertion or data deletion. Two support thresholds are used to, respectively, find the large and prelarge itemsets for reducing the time of rescanning original database. The algorithms are unnecessary to rescan the original database until a number of transactions have been inserted [20, 21], deleted [22], or modified [23]. Since rescanning procedure requires much time, the maintenance cost of rescanning time could thus be saved. When some transactions are inserted into a database, nine cases then arise in Figure 1 [17].

From Figure 1, Cases 1, 5, 6, 8, and 9 do not disturb the final frequent itmesets. Cases 2 and 3 may remove some existing frequent itmesets, and Cases 4 and 7 may generate new frequent itmesets. If all large and prelarge itemsets were kept in the original database, Cases 2, 3, and 4 can be easily handled. Besides, in the maintenance process, the ratio of new transactions compared to old database is usually very small. This is more obvious when database grows large that an itemset in Case 7 cannot possibly be large (frequent) for entire updated database as long as the number of inserted transactions is smaller than the safety number *f* shown below [17]:

(1) $$\begin{array}{c} f=\left\lfloor \frac{\left(S_{u}-S_{l}\right)\times d}{1-S_{u}}\right\rfloor , \end{array}$$

where *f* is the safety number of the new transactions, *S*_*u*_ is the upper threshold, *S*_*l*_ is the lower threshold, and *d* is the number of transactions in original database. A summary of the nine cases and their merged results is shown in Table 1.

### 2.3. Data Sanitization

Data mining techniques can pose security problems and lead to privacy concerns [24] when the discovered information is misused. PPDM techniques have thus become a critical research issue for hiding confidential or secure information. In the past years, Atallah et al. first proposed the protection algorithm for data sanitization to avoid the appearance of the sensitive association rules [25]. It uses both addition and deletion methods to modify databases for hiding sensitive information. Dasseni et al. proposed a hiding algorithm based on the Hamming-distance approach to reduce the confidence and support values of association rules [26]. Three heuristic hiding approaches were proposed to, respectively, increase the support of antecedent parts, to decrease the support of consequent parts, and to decrease the support of either the antecedent or the consequent parts. When the support or the confidence of sensitive association rules is below minimum support thresholds, the sensitive rules are hidden. Hong et al. proposed a SIF-IDF algorithm to sanitize the database for hiding sensitive itemsets through transaction deletion [27]. Lin et al. then proposed a heuristic approach for inserting dummy transactions to greedy hide the sensitive itemsets [28].

The privacy preserving utility mining (PPUM) is an extension of PPDM, considering the utility factors as quantities and profits in real-world applications. In the past, Yeh and Hsu first proposed the protection algorithm for data sanitization to avoid exposing the sensitive information [13]. They proposed two algorithms to modify the quantity of items for hiding the user-specified sensitive information. In the first HHUIF algorithm, the most influential item in the transaction which contains the sensitive high utility itemsets is chosen. The quantity of the chosen item is thus decreased for hiding the sensitive high utility itemsets. In the second MSICF algorithm, the conflict count is considered to find the better choice for deletion. When the itemset utility ratios of sensitive high utility itemsets were below user-specified minimum high utility threshold, the sensitive high utility itemsets could thus be hidden.

In PPUM, the data sanitization for hiding the sensitive information could be divided into two types, which are the item sanitization and the transaction sanitization. The transaction sanitization method is adopted in this paper to protect the sensitive information.

### 2.4. Genetic Algorithms

For the past half century, some nature-inspired algorithms were then proposed to solve the optimization problems, and one of the mostly common ones is genetic algorithms (GAs) [4, 26]. Based on the user-specified fitness function, GAs are thus used to provide the feasible solutions in solving difficult optimization problems. In the past ten years, GAs have been successfully applied to the fields of optimization [9, 11], machine learning [11], and neural networks [10], among others. According to the principle of survival of the fittest, GAs generate the next population by various operations with each individual in the population representing a possible solution. Three main operations used in the genetic algorithm are described below.

*Crossover.* The offspring is generated from two chosen individuals in the population by swapping some bits in the two individuals. The generated offspring thus inherits some characteristics from the two individuals.

*Mutation.* One or several bits of an offspring may be randomly changed. The offspring may thus possess different characteristics from their parents. Mutation increases the possibility to achieve the global optima.

*Selection.* Some excellent offspring are chosen for survival according to predefined rules. This operation keeps the population size within a fixed amount and preserves the good offspring into next generation with a high possibility.

On applying the GAs to find the optimal solution, the first step is to define a representation of the possible solution. The most common way of representation is the bit string. An initial population of *individuals*, called *chromosomes*, is defined as a set of possible solutions. Three genetic operations (crossover, mutation, and selection) are then performed to generate the next generations. Each chromosome is evaluated by a fitness function, which is predefined by user to determine the goodness of chromosome.

**Figure 2:**
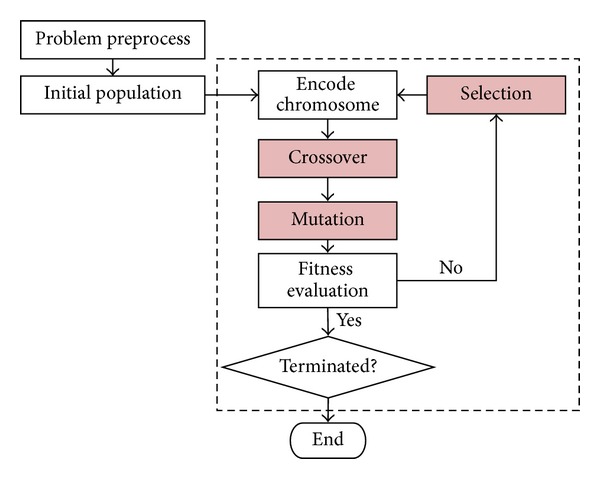
The entire flowchart of the genetic algorithm.

This procedure is recursively performed until the termination criterion is satisfied. In this paper, the single-point *crossover* is used to generate new offspring; the adopted *mutation* operator will change the gene value in a chromosome from one transaction ID to another; a hybrid *selection* method is adopted to combine both the Elitism approach and the Rank approach. The chromosomes in the population are firstly sorted according to their fitness values in ascending order. The top *b* (usually *n*/2) chromosomes are then selected from the new generation with the next (*n* − *b*) chromosomes being randomly selected from the original database forming the next population. Note that the value *n* is initially set as the population size for evaluation. The entirely GA process is shown in Figure 2.

## 3. The GA-Based Approach for Privacy Preserving Utility Mining through Transaction Insertion

In this paper, a GA-based privacy preserving utility mining approach is thus designed to find the appropriate transactions from original database to be inserted in the database. It firstly finds a recommendable size of transactions as the number of genes in a chromosome, which can be determined by the maximal inserted utility value and the total utility value in the original database. The designed algorithm is to extract the transactions from the original database as the optimal solution for a chromosome to be inserted into the database. A lower utility threshold is also obtained to find the prelarge transaction-weighted utilization itemsets, thus avoiding the rescanning time of original database.

A flexible evaluation function with three main factors is also designed in the proposed approach, and different weights may be respectively assigned to them depending on users. Besides, the prelarge concepts with two thresholds and a proposed *sliding count *is used to reduce the rescanning time of the original database, thus enhancing the performance in evaluation process. The details of the algorithm are stated below.

### 3.1. Representation of Chromosomes

In the proposed GA-based approach, a chromosome corresponds to a possible solution to hide the sensitive high utility itemsets in privacy preserving utility mining. A maximal size *m* of chromosome can be discovered from the original quantitative database. The genes in a chromosome can be represented as a possible solution (transaction ID) to be inserted into the database. Note that the genes of a chromosome can contain zero value in the evaluation process.

### 3.2. Fitness Function

**Figure 3:**
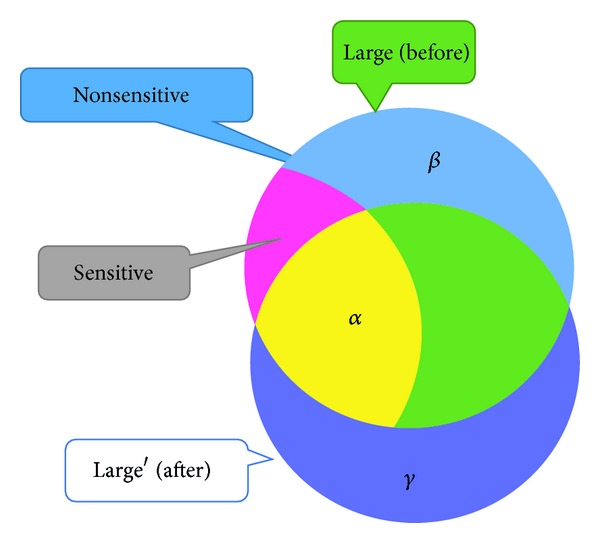
The relationship between itemsets before and after the PPUM process.

In traditional GA-based approaches, it is necessary to define the fitness functions for evaluating the goodness of chromosomes. In privacy preserving utility mining (PPUM) [13], the purpose is to hide the sensitive high utility itemsets with the minimal side effects. The relationship between itemsets before and after the PPUM process can be described in Figure 3, where *H* represents the high utility itemsets in the original database, *S* represents the sensitive high utility itemsets defined by users that are of high utility but need to be protected for privacy preserving, ~*S* represents the nonsensitive high utility itemsets that are of high utility, and *H*′ is the high utility itemsets after the sanitization process for transaction insertion.

Let *α* be the number of sensitive high utility itemsets that fail to be hidden; that is, it is the number of sensitive high utility itemsets that still appear after the sanitization process. In the optimal situation, the value should be zero for totally hiding the sensitive itemsets after PPUM. Thus, *α* can be represented as the interaction of *S* and *H*′( = *S*∩*H*′). To totally hide the sensitive information, *α* should become 0.

Another evaluation criterion is the number of missing high utility itemsets, which is denoted as *β*. A missing high utility itemset is a nonsensitive high utility itemset in the original database but is not discovered from the sanitized database. Thus, *β* can be represented as the difference of ~*S* and *H*′ ( = ~*S* − *H*′). Ideally, the value *β* should be minimized as well after the PPUM.

The last evaluation criterion is the number of artificial high utility itemsets, which is denoted by *γ*. It represents the set of high utility itemsets appearing in the sanitized database but not belonging to the high utility itemset in the original database. Thus, *γ* can be represented as the difference of *H*′ and *H* ( = *H*′ − *H*). Thus, the optimal solution is to minimize the *γ* value for data sanitization.

From the above description, it is known that *α* = *S*∩*H*′, *β* = ~*S* − *H*′ = (*H* − *S*) − *H*′, and *γ* = *H*′ − *H*. The fitness function used in this paper may be defined as follows:

(2) $$\begin{array}{c} \text{fitness}\left(i\right)=w_{1}\times \alpha +w_{2}\times \beta +w_{3}\times \gamma , \end{array}$$

where *w*_1_, *w*_2_, and *w*_3_ are the weighting parameters given by users. In the traditional GA-based approaches for evaluating the fitness value, the original database is required to be rescanned for calculating three factors. This evaluation process requires more computational time to achieve the optimal solution. In the designed GA-based algorithm, the fitness values can be easily evaluated without rescanning database based on the prelarge concept [17] and two-phase approach [16] for firstly deriving the prelarge transaction-weighted utilization itemsets. The discovered itemsets can thus reduce the movement directly from large to small and vice versa when transactions are inserted.

### 3.3. Sliding Count

Prelarge transaction-weighted utilization itemset is not really large (high) transaction-weighted utilization itemset, but it is likely to be the large (high) transaction-weighted utilization itemset in the future through the transaction insertion. Based on the prelarge concept, it is unnecessary to rescan the original database until a number of transactions have been inserted. Since rescanning procedure requires much time, the maintenance cost of rescanning time could thus be reduced.

**Figure 4:**
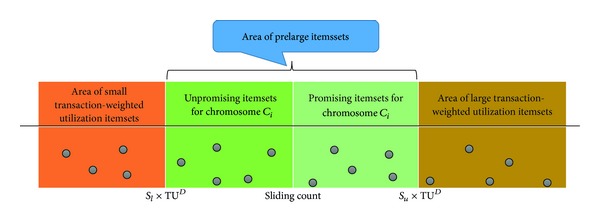
The relation between upper threshold, sliding count, and lower threshold.

For the proposed GA-based algorithm, a maximal utility value for insertion will be obtained to derive the lower utility threshold. Since the lower utility threshold is obtained according to the maximal insertion utility, it can be considered as an overestimated threshold from all of chromosomes. For each chromosome *C*_*i*_, a precise threshold value will be determined according to the insertion utility, which is called *sliding count* for filtering the unpromising prelarge transaction-weighted utilization itemsets to reduce the evaluation cost, which is represented as

(3) $$\begin{array}{c} \text{Sliding}\_\text{Count}=S_{u}\times \left({\text{TU}}^{D}+{\text{TU}}_{i}^{d}\right)-{\text{TU}}_{i}^{d}, \end{array}$$

where TU^*D*^ is the total utility of the original database *D*, TU_*i*_^*d*^ is the total inserted or deleted utility chosen by the chromosome *C*_*i*_, and *S*_*u*_ is the minimal high utility threshold. The relation between upper threshold, sliding count and lower threshold is stated in Figure 4.

### 3.4. Notation

The notations used in the proposed GA-based algorithm are described below:

 
*I*: a set of *r* items, *I* = {*i*_1_, *i*_2_, …, *i*_*j*_, …, *i*_*r*_}, in which each item *i*_*j*_ has its own profit value *p*_*j*_;
 
*P*: the profit table, {*p*_1_, *p*_2_,…, *p*_*j*_, …, *p*_*r*_}, in which *p*_*k*_ is the profit value of an item *i*_*j*_;
 
*D*: the original quantitative database, *D* = {*T*_1_, *T*_2_, …, *T*_*k*_,…, *T*_*v*_}, in which each transaction contains several items with its purchased quantities;
 
*d*: the set of inserted transactions which is chosen from the Cand_Trans, *d* = {*t*_1_, *t*_2_, …, *t*_*j*_, …, *t*_*m*_}, in which *m* is the maximal size of inserted transactions;
 TID: the unique transaction identification for each transaction;
 TU^*D*^: the total utility of the transactions in *D*;
 
*q*_*kj*_: the quantity of item *i*_*j*_ in the transaction *T*_*k*_;
 
*u*_*kj*_: the utility of item *i*_*j*_ in the transaction *T*_*k*_, which is calculated as *q*_*kj*_ × *p*_*j*_;
 tu_*k*_: the transaction utility of transaction *T*_*k*_ or *t*_*k*_, respectively, for the original database and inserted transactions;
 
*S*_*u*_: the upper utility threshold for large (high) transaction-weighted utilization itemsets. It is the same as the high utility threshold in the traditional utility mining;
 
*S*_*l*_: the lower utility threshold for the prelarge transaction-weighted utilization itemsets, *S*_*u*_ > *S*_*l*_;
 Cand_Trans: the set of candidate transactions for insertion;
 
*m*: the size of inserted transaction which is represented as the length of chromosome;
 
*n*: the population size for the GA-based algorithm;
 
*Max*⁡_iutil_: the maximal inserted utility decided by the chromosome size and the content of original database;
 TU_*i*_^*d*^: a chromosome composed by a set of TID;
 
*SC*_*i*_: the total utility of the inserted transactions *d* in the chromosome *C*_*i*_;
 SI: a utility value which is derived for pruning the unpromising prelarge transaction-weighted utilization itemsets in the evaluation process;
 STUB_*p*_: a set of sensitive high utility itemsets SI = {si_1_, si_2_, …, si_*p*_, …, si_*s*_};
 MSTUB: a utility value which represents the safety transaction utility bound for successfully hiding the sensitive high utility itemsets si_*p*_;
 au_*p*_: the maximum safety transaction utility bound;
 IT: an appropriate set of TID to be inserted for privacy preserving.

### 3.5. Proposed Algorithm

*Input.* It includes a quantitative transaction dataset *D*, a minimum high utility threshold *S*_*u*_ (the same as the upper utility threshold), a set of user-defined sensitive high utility itemsets SI, and a population size *n*.

*Output.* It is a set of appropriate transactions IT to be inserted.

**Step 1**

Derive the item utility value *u*_*kj*_ of each item *i*_*j*_ in the transaction *T*_*k*_ as

(4) $$\begin{array}{c} u_{kj}=q_{kj}\times p_{j}, \end{array}$$

where *q*_*kj*_ is the quantity of *i*_*j*_ in *T*_*k*_ and *p*_*j*_ is the profit of *i*_*j*_ in the profit table *P*; sum up the utility values of all the items in each transaction *T*_*k*_ as the transaction utility tu_*k*_ by

(5) $$\begin{array}{c} {\text{tu}}_{k}=\sum_{j=1}^{m}u_{kj}; \end{array}$$

add the transaction utilities for all transactions in *D* as the total utility TU^*D*^ of *D* by

(6) $$\begin{array}{c} {\text{TU}}^{D}=\sum_{k=1}^{n}{\text{tu}}_{k}. \end{array}$$

**Step 2**

Scan database to calculate the actual utility au_*p*_ of each sensitive high utility itemset si_*p*_ as

(7) $$\begin{array}{c} {\text{au}}_{p}=\sum_{T_{k}\in D,T_{k}⊇{\text{si}}_{p},i_{j}\in {\text{si}}_{p}}u_{kj}. \end{array}$$

**Step 3**

Calculate the maximum safety transaction utility bound (MSTUB) for newly inserted transactions as

(8) $$\begin{array}{c} \text{MSTUB}=\overset{s}{\underset{p=1}{\max}}\left({\text{STUB}}_{p}\right)=\overset{s}{\underset{p=1}{\max}}\left(\frac{{\text{au}}_{p}}{S_{u}}-{\text{TU}}^{D}\right), \end{array}$$

where STUB_*p*_ is the safety transaction utility bound of the sensitive high utility itemset si_*p*_, TU^*D*^ is the total utility of the original database *D*, and au_*p*_ is the actual utility of the sensitive high utility itemset si_*p*_.

**Step 4**

Extract the transactions from the original database *D* without containing any sensitive high utility itemsets SI as the candidate transactions for insertion. Put the extracted TID in the set of Cand_Trans.

**Step 5**

Sort the extracted transactions in the set of Cand_Trans in ascending order by their transaction utility tu_*k*_.

**Step 6**

Sum up the sorted transactions by their transaction utility tu_*k*_ tuple by tuple; terminate the summation process until the summed value is larger than to the maximal safety transaction utility bound MSTUB; calculate the number of transactions *m* to be terminated under the above condition, in which *m* is used as the size of chromosome in this GA-based algorithm.

**Step 7**

Sum up the top-*m* transaction utility tu_*k*_ in sorted transactions as the maximal inserted utility *Max*⁡_iutil_.

**Step 8**

Derive the lower utility threshold *S*_*l*_ as

(9) $$\begin{array}{c} S_{l}=S_{u}-\frac{{\mathrm{Max}}_{\text{iutil}}}{{\text{TU}}^{D}}\times \left(1-S_{u}\right). \end{array}$$

**Step 9**

Scan database to find the large (high) transaction-weighted utilization itemsets and the prelarge transaction-weighted utilization itemsets based on the upper utility threshold *S*_*u*_ and the lower utility threshold *S*_*l*_, respectively.

**Step 10**

Initially generate a population of *n* individuals with *m* genes randomly. Each gene is represented as the transaction identification TID chosen from the set of Cand_Trans.

**Step 11**

Execute the crossover operations on the population.

**Step 12**

Execute the mutation operations on the population.

**Step 13**

Calculate the fitness value of each chromosome *C*_*i*_ in the population. Do the following substeps.

*Step  13.1*. Sum up the transaction utility of each chromosome *C*_*i*_ as TU_*i*_^*d*^.

*Step  13.2*. If TU_*i*_^*d*^ is larger than the maximal safety transaction utility bound MSTUB, calculate the sliding count *SC*_*i*_; otherwise, the chromosome is considered as an illegal composition, and do Step  13.4. Calculate the sliding count *SC*_*i*_ as

(10) $$\begin{array}{c} SC_{i}=S_{u}\times \left({\text{TU}}^{D}+{\text{TU}}_{i}^{d}\right)-{\text{TU}}_{i}^{d}, \end{array}$$

where TU^*D*^ is the total utility of the original database *D*, TU_*i*_^*d*^ is the total inserted utility chosen by the chromosome *C*_*i*_, and *S*_*u*_ is the minimal high utility threshold.

*Step  13.3*. Update the large (high) transaction-weighted utilization itemsets and prelarge transaction-weighted utilization itemsets for each chromosome *C*_*i*_ by upper utility count (*S*_*u*_ × TD^*D*^) and *SC*_*i*_ value, respectively.

*Step  13.4*. Calculate the fitness value as

(11) $$\begin{array}{c} \text{fitness}\left(i\right)=\{\begin{array}{cc} \infty , & {\text{TU}}_{i}^{d}\leq \text{MSTUB},\\ w_{1}\times \alpha +w_{2}\times \beta +w_{3}\times \gamma , & \text{Otherwise}, \end{array} \end{array}$$

where *w*_1_, *w*_2_, and *w*_3_ are weighting parameters which are defined by user, *α* is the number of sensitive high utility itemsets that fail to be hidden, *β* is the number of missing high utility itemsets, and *γ* is the number of artificial high utility itemsets.

**Step 14**

Choose the top *b* chromosomes from the population and randomly select (*n* − *b*) chromosomes from the original database to generate the *n* chromosomes in the next population.

**Step 15**

If the termination criterion is not satisfied, go to Step 11; otherwise, do the next step.

**Step 16**

Output the inserted transaction identifications IT as the best chromosome to users.

## 4. An Illustrated Example

In this section, an example is given to illustrate the proposed GA-based algorithm for privacy preserving utility mining. Assume an original quantitative database includes 12 transactions, which is shown in Table 2. Each transaction consists of its transaction identification (TID), items with its purchased quantities. The profit of each item is then shown in Table 3 as the utility table.

The minimum high utility threshold *S*_*u*_ is set at 30%. Based on the two-phase approach [16] for mining high utility itemsets, the discovered high utility itemsets in the original database are {*C*}, {*D*}, {*BD*}, and {*BCD*}. Suppose that the sensitive high utility itemsets are defined as {*D*} and {*BCD*}. The proposed algorithm is then performed as follows.

**Step 1**

The transaction utility value of each item occurring in each transaction in Table 2 is first calculated. Take the first transaction as an example to illustrate the steps. Since there is one item in the first transaction, the transaction utility for the first transaction in Table 2 is thus calculated as tu_1_ = 6 × 6 = 36. The transaction utilities for the other transactions in Table 2 are also calculated in the same way. The results are then shown in Table 4. The total utility of all transactions in Table 4 is thus calculated as (36 + 37 + 12 + 75 + 80 + 72 + 30 + 8 + 98 + 10 + 75 + 27), which is 560.

**Step 2**

The actual utility value of each sensitive high utility itemset is first calculated. Take the first sensitive high utility itemset {*D*} as an example to illustrate the steps. The sensitive high utility itemset {*D*} appears in transactions 2, 7, 9, 11, and 12 with quantities which are, respectively, 5, 4, 7, 5, and 3 which is shown in Table 2. The profit of item *D* is 7 in Table 3. The actual utility of au^*d*^(*D*) is thus calculated as (5 + 4 + 7 + 5 + 3) × 7 ( = 168). Another sensitive high utility itemset {*BCD*} is performed in the same way. The results are then shown in Table 5.

**Step 3**

The safety bound of each sensitive high utility itemset is represented as the minimal utility value for transaction insertion to hide the sensitive high utility itemset. Take the sensitive high utility itemset {*D*} as an example to illustrate the steps. The safety bound of sensitive high utility itemset {*D*} is thus calculated as (au_*p*_/*S*_*u*_) − TU^*D*^( = 168/0.3) − 560( = 0), where au_*p*_ is the actual utility of sensitive high utility itemset {*D*}, *S*_*u*_ is the upper utility threshold, and TU^*D*^ is the transaction utility in the original database. Another sensitive high utility itemset {*BCD*} is processed in the same way. The results are then shown in Table 6. Thus, the maximum safety transaction utility bound MSTUB is considered as 16.67 for the two sensitive high utility itemsets.

**Step 4**

For each transaction in Table 4, if the transaction is not containing any sensitive high utility itemset, the transaction identification TID is then put into the set of Cand_Trans. Take the first transaction in Table 4 as an example to illustrate the processes. Since the first transaction does not contain any of sensitive high utility itemsets, it is thus considered as the candidate transaction for transaction insertion. The other transactions in Table 4 are also processed in the same way. After that, the remaining candidate transactions for transaction insertion are then shown in Table 7.

**Step 5**

The transactions in Table 7 are then sorted in ascending order according to their transaction utilities. The results are shown in Table 8.

**Step 6**

The sorted transactions in Table 8 are then accumulated tuple by tuple until the summed value of transaction utility is larger than the maximum safety transaction utility bound MSTUB( = 16.7), which is calculated as (8 + 10)( = 18) > 16.67. The number of transactions is thus achieved by the above condition and set at 2, which is used for the number of genes in a chromosome in the proposed algorithm.

**Step 7**

Sum up the top-2 transaction utility values as the maximal inserted utility for the top-2 inserted transactions, which is calculated as *Max*⁡_iutil_( = 80 + 75)( = 155).

**Step 8**

The lower utility threshold (*S*_*l*_) can be obtained according to the formula *S*_*l*_ = *S*_*u*_ − (*Max*⁡_iutil_/TU^*D*^)×(1 − *S*_*u*_). The lower utility threshold is then calculated as 0.3 − (155/560)×(1 − 0.3), which is 10.62%.

**Step 9**

After the lower threshold is obtained, the original database is then scanned to, respectively, get the large and the prelarge transaction-weighted utilization itemsets with their transaction-weighted utilization values and actual utility values according to the upper utility threshold *S*_*u*_( = 30%) and lower utility threshold *S*_*l*_( = 10.62%). The large transaction-weighted utilization itemsets and the prelarge transaction-weighted utilization itemsets are then shown in Tables 9 and 10.

**Step 10**

A population of 1000 individuals (chromosomes) with two genes composed by TID is randomly generated from the set of the candidate transactions appearing in Table 7. In this example, each chromosome is composed of two genes. Assume that a possible composition is *T*_1_, and *T*_10_ are randomly selected from the candidate transactions in the set of Cand_Trans to form an initial chromosome. Thus, the chromosome is considered a legal composition and represented as (1, 10).

**Step 11**

The crossover operation is executed on the population.

**Step 12**

The mutation operation is executed on the population.

**Step 13**

Assume a chromosome (3,10) is obtained as a possible solution. The sliding count for this chromosome is calculated as (560 + 22) × 0.3 − 22 ( = 152.6), which is used to filter the unpromising prelarge itemset to reduce the computation time. The evaluation process is then performed for the itemset with its transaction-weighted utilization being larger than or equal to the sliding count. After filtering, the results are shown in Table 11.

**Figure 5:**
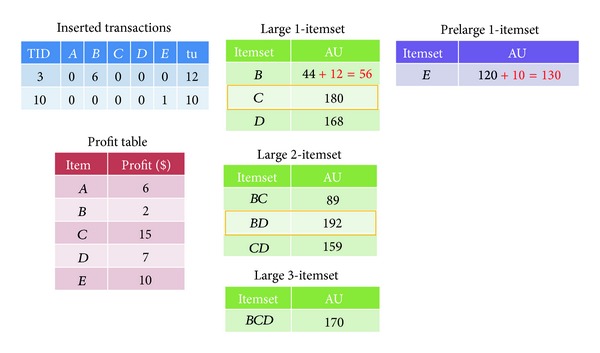
The set of sensitive high utility itemsets that fail to be hidden.

With the aid of the prelarge transaction-weighted itemsets, it easily derives the required rules without rescanning database. In this example, the itemsets in Tables 9 and 11 are then updated. After that, the results are shown in Figure 5. The final high utility itemsets for the chromosome (3, 10) are thus {*C*}.

After the high utility itemsets shown in Table 9 are obtained, the number *α* of sensitive high utility itemsets that fail to be hidden, the number *β* of missing high utility itemsets, and the number *γ* of artificial high utility itemsets can be easily evaluated. In the above example, the set of sensitive itemsets that fail to be hidden is {*D*, *BCD*}∩{*C*, *BD*}, which is *Ø*. The value of *α* is thus 0. The set of missing itemsets is {*C*, *D*, *BD*, *BCD*}−{*D*, *BCD*}−{*C*, *BD*}, which is *Ø*. The value of *β* is thus 0. The set of artificial itemsets is {*C*, *BD*}−{*C*, *D*, *BD*, *BCD*} = *Ø*. The value of *γ* is thus 0. Let the three weight parameters be set as 0.5, 0.25, and 0.25, respectively. The fitness value of the chromosome is then calculated as follows:

(12) $$\begin{array}{c} \text{fitness}=0.5\times 0+0.25\times 0+0.25\times 0=0. \end{array}$$

The other *chromosomes* in the population are evaluated in the same way to find the feasible solution for PPUM.

**Step 14**

In this step, *b* is set at *n*/2( = 1000/2)( = 500). Thus, the top 500 chromosomes from the current population and the randomly chosen (*n* − *b*)( = 1000 − 500) ( = 500) chromosomes from the original database are merged together to form the next population for generation.

**Step 15**

In the example, two criteria are used as the termination condition. The first condition is when the fitness value is achieved in stable situation. In this example, the best fitness value is set at 0 without any side effects. Another condition to terminate the progress is when the number of generations is achieved by the user-defined number.

**Step 16**

Finally, the best composition of a chromosome by the transaction identification TID to be inserted is thus selected as the best solution to users.

## 5. Experimental Results

The experiments are implemented in Java language and executed on a PC with 3.0 GHz CPU and 4 GB memory. Two datasets are used in the experiments, which are, respectively, from IBM data generator for a simulation dataset [29] and the foodmart database [30] in the real-world applications. A simulation model [16] is then developed to generate the quantities of the items in the transactions for database, which is generated from IBM data generator. The range of quantity is set from 1 to 5 and randomly from 0.01 to 10 for profit in the utility table, respectively. The foodmart dataset is collected from an anonymous chain store composed by a quantitative database about the products sold by the chain store. There are 21,556 transactions and 1,559 items in the dataset.

**Figure 6:**
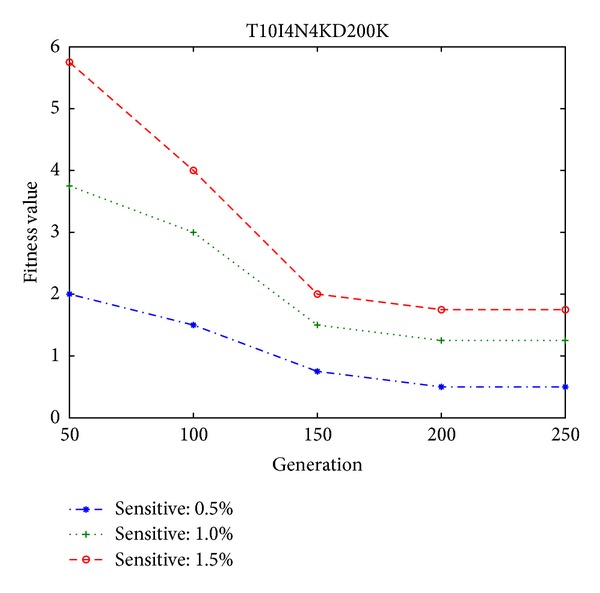
Fitness values in different percentages of sensitive high utility itemsets in the simulation dataset.

**Figure 7:**
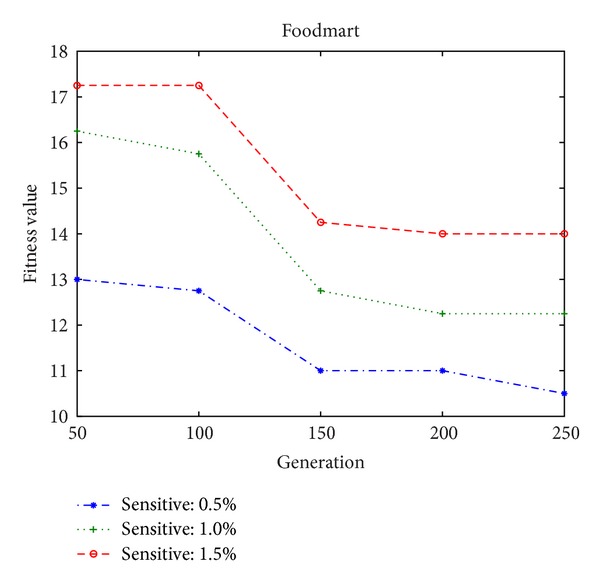
Fitness values in different percentages of sensitive high utility itemsets in the foodmart database.

In the experiments, the minimum high utility thresholds are, respectively, set at 0.2% and 0.02% for both the simulation dataset and the foodmart database. The numbers of sensitive high utility itemsets are then defined by the percentages of the high utility itemsets in the database, which can obviously show the performance of the proposed algorithm. The population size of the genetic algorithm is initially set at 100. The fitness values are firstly compared among different percentages of sensitive high utility itemsets in two different databases, respectively, shown in Figures 6 and 7, which is used to show the convergence rates and the fitness value (summed up number of each side effect × its adjustable weight) of the proposed approach.

**Figure 8:**
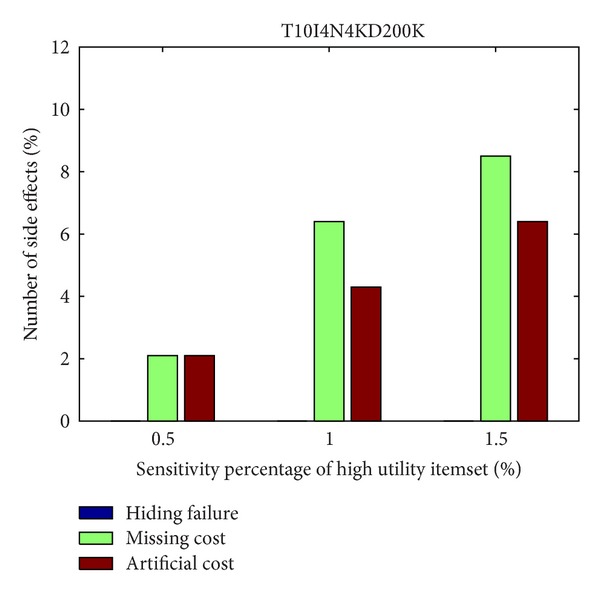
The evaluation of three side effects in simulation dataset.

**Figure 9:**
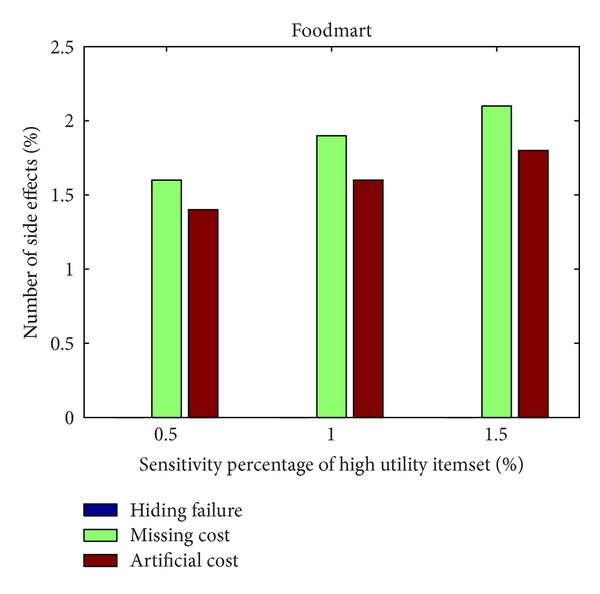
The evaluation of three side effects in foodmart database.

From Figures 6 and 7, it can be observed that the designed algorithm can thus achieve a stable situation with the growing generations. The side effects of the proposed GA-based approach are then evaluated, including the hiding failure for the number of sensitive high utility itemsets, the number of the missing high utility itemsets, and the number of artificial high utility itemsets, respectively, shown in Figures 8 and 9, in two databases. This evaluation criteria are used to find out the generation of the number of side effects by the proposed algorithm.

**Figure 10:**
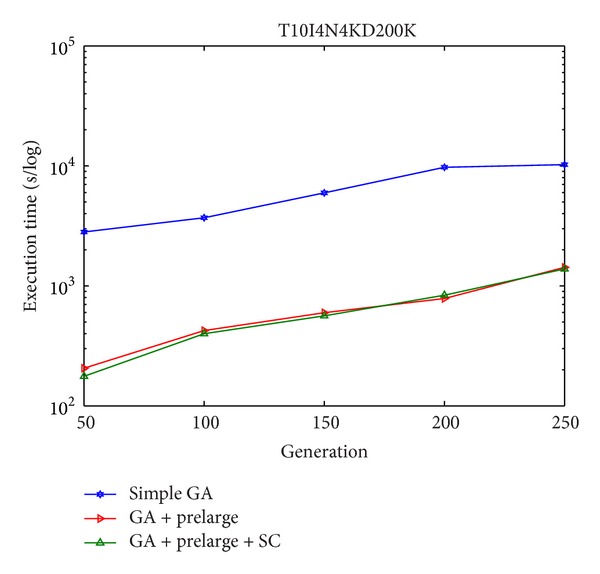
Execution time of the original GA-based algorithm and the GA-based algorithm involved prelarge concept (with and without sliding count) in simulation dataset.

**Figure 11:**
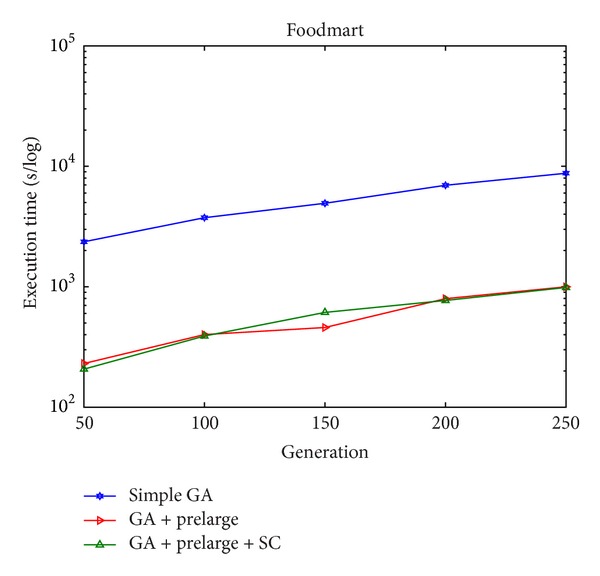
Execution time of the original GA-based algorithm and the GA-based algorithm involving prelarge concept (with and without sliding count) in foodmart database.

In Figures 8 and 9, it can be seen that there are only few side effects of hiding failure in different percentages of sensitive high utility itemsets by the proposed GA-based algorithm. The sensitive high utility itemsets are totally hiding in the proposed approach. The difference between the GA-based algorithm and the GA-based algorithm involving the prelarge concepts is then also compared, respectively, in the simulation dataset and foodmart database. The results are then shown in Figures 10 and 11. This evaluation criteria are used to find the performance of the execution time compared to the simple genetic algorithm with the proposed GA-based approach based on prelarge concept (with and without sliding count).

From Figures 10 and 11, it is obvious to see that the proposed GA with the prelarge concepts can greatly reduce the computational time for rescanning the original database compared to the simple GA-based algorithm. Besides, the sliding count has a slight help for reducing the execution time.

## 6. Conclusions and Future Works

In this paper, a GA-based algorithm is proposed to find the feasible combination for data sanitization. Each gene in a chromosome represents a possible transaction to be inserted. Three user-specific weights are thus assigned to three factors, which are hiding failure sensitive high utility itemsets, missing high utility itemsets, and artificial high utility itemsets, to evaluate the fitness values of chromosomes. Besides, the designed sliding count based on prelarge concepts is also applied to the GA-based approach, thus reducing the computational time of rescanning database.

In the proposed GA-based algorithm, transaction insertion is used to hide the sensitive high utility itemsets based on prelarge concepts. The prelarge concepts consisted of insertion, deletion, and modification. How to combine those concepts to produce the dummy transactions for PPUM is another critical issue in the future.

## Figures and Tables

**Table 1:** Nine cases and their results for transactions insertion based on the pre-large concepts.

Cases	Original/new	Merged results
Case 1	Large/large	Large

Case 2	Large/prelarge	Large or prelarge, decided through existing information

Case 3	Large/small	Large, prelarge, or small, decided through existing information

Case 4	Prelarge/large	Large or prelarge, decided through existing information

Case 5	Prelarge/prelarge	Prelarge

Case 6	Prelarge/small	Prelarge or small, decided through existing information

Case 7	Small/large	Prelarge or small, when the number of transactions is small

Case 8	Small/prelarge	Prelarge or small

Case 9	Small/small	Small

**Table 2:** An originally quantitative database.

TID	*A *	*B *	*C *	*D *	*E *
1	6	0	0	0	0
2	0	1	0	5	0
3	0	6	0	0	0
4	0	0	5	0	0
5	0	0	0	0	8
6	2	0	2	0	3
7	0	1	0	4	0
8	0	4	0	0	0
9	0	2	3	7	0
10	0	0	0	0	1
11	0	5	2	5	0
12	0	3	0	3	0

**Table 3:** A proit table.

Item	Profit ($)
*A*	6
*B*	2
*C*	15
*D*	7
*E*	10

**Table 4:** Transaction utilities.

TID	*A*	*B*	*C*	*D*	*E*	Transaction utility
1	6	0	0	0	0	36
2	0	1	0	5	0	37
3	0	6	0	0	0	12
4	0	0	5	0	0	75
5	0	0	0	0	8	80
6	2	0	2	0	3	72
7	0	1	0	4	0	30
8	0	4	0	0	0	8
9	0	2	3	7	0	98
10	0	0	0	0	1	10
11	0	5	2	5	0	75
12	0	3	0	3	0	27

**Table 5:** Actual utilities for the sensitive high utility itemsets.

Sensitive high utility itemset	Actual utility
{*D*}	168
{*BCD*}	173

**Table 6:** Safety bounds for the sensitive high utility itemsets.

Sensitive high utility itemset	Safety bound
{*D*}	0
{*BCD*}	16.67

**Table 7:** The candidate transactions for insertion with their transaction utilities.

TID	*A*	*B*	*C*	*D*	*E*	Transaction utility
1	6	0	0	0	0	36
3	0	6	0	0	0	12
4	0	0	5	0	0	75
5	0	0	0	0	8	80
6	2	0	2	0	3	72
8	0	4	0	0	0	8
10	0	0	0	0	1	10

**Table 8:** The sorted candidate transactions for insertion.

TID	*A*	*B*	*C*	*D*	*E*	Transaction utility
8	0	4	0	0	0	8
10	0	0	0	0	1	10
3	0	6	0	0	0	12
1	6	0	0	0	0	36
6	2	0	2	0	3	72
4	0	0	5	0	0	75
5	0	0	0	0	8	80

**Table 9:** The large transaction-weighted utilization itemsets.

Large 1-itemset			Large 2-itemset			Large 3-itemset		
Itemset	TWU	AU	Itemset	TWU	AU	Itemset	TWU	AU
{*B*}	287	44	{*BC*}	173	89	{*BCD*}	173	173
{*C*}	320	180	{*BD*}	267	192			
{*D*}	267	168	{*CD*}	173	159			

**Table 10:** The pre-large transaction-weighted utilization itemsets.

Pre-large 1-itemset			Pre-large 2-itemset			Pre-large 3-itemset		
Itemset	TWU	AU	Itemset	TWU	AU	Itemset	TWU	AU
{*A*}	108	48	{*AC*}	72	42	{*AC* *E*}	72	72
{*E*}	162	120	{*AE*}	72	42			
			{*CE*}	72	60			

**Table 11:** The filtered pre-large transaction-weighted utilization itemsets by sliding count.

Pre-large 1-itemset		
Itemset	TWU	AU
{*E*}	162	120
